# Building the Older Adult Fall Prevention Movement – Steps and Lessons Learned

**DOI:** 10.3389/fpubh.2014.00194

**Published:** 2015-04-27

**Authors:** Ellen C. Schneider, Bonita Lynn Beattie

**Affiliations:** ^1^Center for Health Promotion and Disease Prevention, University of North Carolina at Chapel Hill, Chapel Hill, NC, USA; ^2^Falls Free^®^ Initiative, National Council on Aging, Washington, DC, USA

**Keywords:** fall prevention, fall prevention movement, Falls^®^ Free Initiative, awareness, advocacy

## Abstract

**Background:**

Falls are the leading cause of older adult injuries and injury-related deaths. Until 2004, the growing public health issue of older adult falls received little national attention. To elevate and focus on the issue, the National Council on Aging launched the Falls Free^®^ Initiative, a group of national and state agencies working collaboratively to address older adult falls with evidence-based solutions. Since then, attention to older adult falls has gained significant momentum.

**Purpose:**

To describe the steps taken to create the momentum around fall prevention and lessons learned that could be applied to supporting other older adult health-related issues.

**Method/objectives:**

The Falls Free^®^ Initiative took key steps to promote the older adult falls prevention movement, including initiating organized advocacy and supporting the development of state coalitions through increasing awareness of the issue, promoting evidence-based programs, instituting evaluation, implementing systems change, and providing tailored technical assistance.

**Results:**

Through the support of the Falls Free^®^ Initiative and many partners, advocacy efforts have increased federal funding for fall prevention, the majority of states have fall prevention coalitions, and thousands of stakeholders are now engaged in fall prevention. Select lessons learned include leveraging compelling data, choosing passionate leaders for the movement, aligning the cause with partner missions, and being inclusive of all stakeholders.

**Conclusion:**

Although much progress has been made in the fall prevention movement, the issue is growing along with the aging population. Efforts must continue to gain support from all affected stakeholders to reduce older adult falls and fall-related injuries.

In 2012, over 2.4 million older adults were treated in emergency departments for falls; more than 722,000 or 30% of these patients had to be hospitalized (1). Every 29 min, an older adult in the United States dies from fall-related injuries (2). Direct medical costs for fall injuries total over $30 billion per year in the nation and account for 6% of all medical expenditures for this age group (3, 4). The risk of falling increases with age, and accelerates after age 85 years due to issues such as declining muscle strength, increased frailty, poor eyesight, and limited movement (5). With an increased life expectancy among the growing baby boomer population, the problem of older adult falls has the potential to overwhelm resources required to address the needs.

Until 2004, the issue of older adult falls received little national attention in part due to its complexity and lack of readily available evidence-base interventions. As a growing public health issue, it clearly needed a national effort to promote awareness and action. Since then, attention to the issue of older adult falls has gained significant momentum through the work of many stakeholders around the country, primarily led through the National Council on Aging’s (NCOA) Falls Free^®^ Initiative (6).

Launched in 2005, the Falls Free^®^ Initiative brought together national and state agencies to collaboratively address older adult falls with evidence-based solutions; the authors of this article were leaders in the effort. The Falls Free^®^ Initiative has been particularly successful in advocacy at the national level and in supporting the creation and development of state fall prevention coalitions and local collaborative efforts across the country. The purpose of this paper is to describe the steps taken to create the momentum around fall prevention and lessons learned that could be applied to supporting other older adult health-related issues. Steps include creating a national initiative; initiating advocacy efforts; and developing and supporting coalitions to increase awareness of the issue, promote evidence-based programs, institute evaluation, and implement policy and systems change.

## Background of the Falls Free^®^ Initiative

In the early 2000s, the Archstone Foundation, a private grantmaking organization based in California, began funding local fall prevention initiatives in the state. The Archstone Foundation was pleased with the growth of statewide activities and the subsequent development of a state fall prevention plan. The Foundation approached the NCOA to design a similar initiative on a national level. NCOA, with funding from the Archstone Foundation and the Home Safety Council, seated an advisory group of leading fall prevention experts to begin planning and conducted an environmental scan of organizations that were or should be working on fall prevention to identify key national stakeholders.

Concurrently, leading researchers were commissioned to develop review papers based on the best available evidence on fall prevention strategies targeted toward community-residing older adults. The review papers focused on the topics of physical mobility, medications management, home safety, environmental safety in the community, and additional cross-cutting areas for attention such as policy and advocacy.

With continued financial support from the Archstone Foundation and Home Safety Council, NCOA convened the national Falls Free^®^ Summit in Washington, DC, USA in December 2004. Fifty-eight national organizations, professional associations, federal agencies, and leading fall prevention experts were invited to participate in this landmark summit to review the evidence and design a national blueprint for reducing falls among older adults.

As a result of the Summit, the National Action Plan (Plan available at http://www.ncoa.org/improve-health/center-for-healthy-aging/content-library/FallsFree_NationalActionPlan_Final.pdf) was created with 9 goals and 36 evidence-based strategies key to reducing older adult falls (7). Goals and strategies were offered for both providers and for older adults corresponding with the review papers on the evidence related to physical mobility, medications management, home and environmental safety, and cross-cutting issues. The long range vision of the Plan was that older adults would have fewer falls and fall-related injuries, maximizing their independence and quality of life (7). More than 8,000 print and CD ROM copies of the Plan were distributed; the Plan was also posted to the NCOA website where it has been downloaded over 125,000 times (personal communication, Emily Dessem, National Council on Aging, 2014 March 2). The purpose of the plan was to develop and enrich supplemental and complementary community-based programs and services to provide a continuum of care aimed at reducing falls and fall-related injuries, not to undermine medical interventions.

When the Plan was released, there was insufficient funding to mount a national campaign to promote action of its 36 strategies. However, in response to the participants’ enthusiasm for the Summit process and the Plan itself, and in an effort to promote the strategies, the Falls Free^®^ Initiative was created (6). This loose-knit collaborative of Summit attendees and their organizations was charged with working toward the progress of one or more of the strategies that resonated with their organizational missions. Since its inception, the Falls Free^®^ Initiative has grown to over 70 national organizations, profession associations, and federal agencies (6).

The original 36 strategies remain relevant and evidence-supported. In 2008, the Falls Free^®^ National Advisory Group convened to review progress made over its 3-year history. Group members engaged in a rich, broad-based exchange of ideas; this deliberation resulted in a number of recommendations and observations across the strategies presented in the National Action Plan, as well as emerging opportunities.

## Initiating Organized Fall Prevention Advocacy

In 2006, the Falls Free^®^ Initiative recognized that the issue of older adult falls needed an active effort to advocate for appropriate national funding levels. Therefore, the National Falls Free^®^ Advocacy Workgroup was formed and successfully advocated for the passage of the Keeping Seniors Safe from Falls Act, signed into law in April 2008 as PL 110-202 (8). The Act enfolded strategies taken directly from the National Action Plan authorizing research, demonstration programs, provider training, and public education to prevent older adult falls. Although the Act passed, no funding was appropriated with its enactment. The Workgroup continued its advocacy efforts and successfully doubled fall prevention funding for the Centers for Disease Control and Prevention’s (CDC) National Center for Injury Prevention and Control (NCIPC) from $1 million in fiscal year 2008 to approximately $2 million in fiscal year 2009 and subsequent years.

Centers for Disease Control and Prevention has used those funds to translate and test evidence-based programs, conduct demonstration projects, and develop the STEADI (Stopping Elderly Accidents, Deaths and Injuries) Tool Kit for health care providers (9). In 2014, continued advocacy efforts led to the allocation of $5 million from the Affordable Act’s Prevention and Public Health Fund for elder fall prevention to the Administration for Community Living/Administration on Aging (AoA) (10). Funds are to increase the availability of and accessibility to effective programs and services in communities.

## Developing State Fall Prevention Coalitions

In 2006, the Falls Free^®^ Initiative accelerated with the addition of the State Coalitions on Fall Prevention Workgroup. At that time, only four states had fall prevention coalitions. These states approached Falls Free^®^ leadership and asked to join the effort. The State Coalitions on Fall Prevention Workgroup was formed and designed to facilitate collaboration between states working on similar issues. The State Coalition Workgroup members reported that developing a state or large regional coalition to address falls and fall-related injuries offered a common forum for multidisciplinary organizations to address falls, deter duplication of efforts, raise awareness, and facilitate necessary roles of resource coordination, policy development, and systems change at the state level (11).

To encourage other states to develop their own fall prevention coalitions, the Falls Free^®^ Initiative developed a tool kit, *Falls and Fall-Related Injuries Among Older Adults: A Practical Guide to State Coalition Building to Address a Growing Public Health Issue* (11). Based on available evidence for coalition building, it was designed to enfold the strategies, experiences, and lessons learned of the 10 fall prevention coalitions in existence by 2007 when the tool kit was created. It includes three stages and nine recommended steps, each with many subtasks, to initiate and build an effective Fall Prevention Coalition. The tool kit still serves as the basis of NCOA technical assistance to states and local communities seeking to build coalitions.

In addition to the tool kit, Falls Free^®^ Initiative leadership provided individualized technical assistance to over 30 states that expressed interest in forming a fall prevention coalition. Technical assistance included structured calls or in-person meetings with coalition leads to walk them through the steps in the Coalition Building tool kit. Support was provided to answer questions about membership sectors, coalition structure, goals and objectives, funding, and evaluation. Formal quarterly calls were also held with the full State Coalitions on Fall Prevention Workgroup to problem solve and collaborate.

As new coalitions formed, they were added to the *State Coalitions on Fall Prevention: Working Collaboratively to Make a Difference Compendium of Initiatives* (12). This document was translated to an interactive map on the NCOA state fall prevention coalition website (13). The website features background and contact information for each coalition so that potential partners can join or have questions addressed about fall prevention activities in the state. Subsequently, NCOA worked with states to develop their own unique state profile of the impact of falls; the states included demonstrate a powerful visual for advocacy purposes (14).

**Figure 1:**
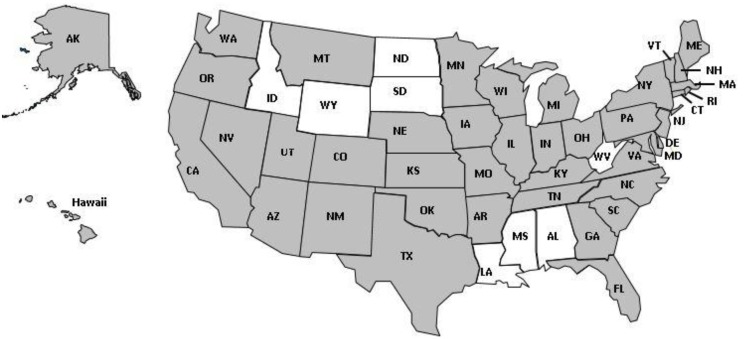
**States with active or developing fall prevention coalitions in 2014**.

In 2007, NCIPC and AoA entered into an interagency agreement to promote evidence-based fall prevention intervention including support of the Falls Free^®^ Initiative. In the same year, NCIPC also named older adult falls as one of its top three priority areas. Making older adult falls a priority helped to engage the public health community and foster the development of fall prevention coalitions in states with CDC Core Violence and Injury Prevention Program grants; that funding could be used to support fall prevention activities and Injury Community Planning Groups with falls as a priority. From 2006 to 2014, the number of active or developing state fall prevention coalitions grew from 4 to 43 (see Figure 1).

## Supporting State Fall Prevention Coalitions

As the number of coalitions grew, the NCOA continually collaborated with them to provide targeted technical assistance and assist them to enhance their individual and collective impact. The following discussion outlines the processes and tools NCOA’s Falls Free^®^ Initiative leadership, with support and input from coalitions and partners, developed to support and sustain their efforts.

### Increasing fall prevention awareness

In 2008, the 10 state-member Falls Free^®^ State Coalitions on Fall Prevention Workgroup (15) requested assistance in declaring a day of awareness; four states had already targeted the autumnal equinox, which NCOA and the remaining states adopted. In response, the National Advocacy Workgroup gained bipartisan sponsorship of the first annual National Falls Prevention Awareness Day (FPAD) resolution in the U.S. Senate and has obtained bipartisan sponsorship every year since then. The number of states observing FPAD grew from 4 in 2007 to 11 in 2008, 22 in 2009, 36 in 2010, 43 in 2011, 46 in 2012, and 47 in 2013, plus the District of Columbia (15).

According to a survey of state fall prevention coalition leads conducted by the NCOA, an estimated 2,076,041 older adults were reached during FPAD activities in 2013, more than 511,000 participated in evidenced-based fall prevention programs, over 17,000 were screened for falls risks, and more than 1.5 million older adults, family caregivers, professionals, and policymakers were reached through advocacy events and education and awareness campaigns (16). Since 2011, NCOA has offered an annual webinar in advance of FPAD to generate creative partnerships and activities across the country. Each year, webinar registration from across the country has exceeded 1,000.

States indicated that increasing awareness of fall prevention was an important goal of their coalitions (11), and several implemented fall prevention awareness campaigns. To better understand the fall prevention awareness campaigns that the states and their fall prevention coalitions implemented and the lessons learned other states could apply to their awareness campaigns, Falls Free^®^ leadership interviewed 10 state agencies and 1 national organization between October 2008 and February 2009. As a result of those interviews, *Falls Prevention Awareness: Lessons Learned from State Coalitions on Fall Prevention* (17) was developed to assist other states in developing fall prevention awareness campaigns. The document contains numerous lessons learned about target audiences, messaging, media and methods, and recommendations for FPAD activities.

### Promoting evidence-based programs

The Falls Free^®^ Initiative is housed within the Center for Healthy Aging (CHA); CHA’s mission is to promote evidence-based health promotion and disease prevention programs. The CHA has been working with a variety of state and local grantees since 2003 to adopt and sustain programs making a difference in the health, independence, and quality of life of older adults. These programs provide measurable improvements in patient outcomes and build patient knowledge skills and confidence to manage problems.

Since providers are increasingly being required by Centers for Medicare and Medicaid (CMS) to promote appropriate healthy behaviors, evidence-based programs can be a valuable asset for provider referrals. In alignment with this requirement, a key strategy of the Falls Free^®^ Initiative is to increase access to quality programs and promote linkages and referrals from the health care community. A number of strategies within the *State Policy Toolkit for Advancing Falls Prevention* (discussed below) promote this effort to affect fall prevention.

There are a variety of evidence-based programs recognized as effective for fall prevention. A *CDC Compendium of Effective Fall Interventions: What Works for Community-Dwelling Older Adults, 2nd Edition* represents a significant CDC investment in providing access to programs that can work in a variety of community and home settings and have been shown through randomized trials to reduce falls (18). However, few of the 22 programs listed offer training, tools, and resources for successful implementation. Programs ready to implement include Stepping On, Tai Chi: Moving for Better Balance and Otago. In addition, ACL/AoA recognizes A Matter of Balance and others available for funding under the Older Americans Act, Title IIID (19).

Ongoing Falls Free^®^ collaborative activities promote sharing among fall prevention coalitions of best practices, strategies, and funding opportunities for evidence-based programs. Active partnership-building strategies are used to link coalitions with national Falls Free^®^ member organizations such as the American Physical Therapy Association through their state chapters and local activities.

### Instituting evaluation

State fall prevention coalitions expressed the need to demonstrate the impact of their coalition work to a variety of stakeholders, coalition partners, funding organizations, and policymakers. In response to those requests, NCOA organized an Evaluation Committee of the State Coalitions on Fall Prevention Workgroup to support state and local evaluation efforts. Members included state coalition leads, researchers, advisors, and CDC staff. The Evaluation Committee developed guidelines to help state fall prevention coalitions evaluate the impact of their efforts, and foster comparisons across states (20). The availability of a standard evaluation process and strategies helps to develop state baseline measures, promote consistency in evaluation efforts across states, and provide data for advocacy or funding opportunities.

The Evaluation Guidelines contain two important products for state coalitions, including the Falls Free^®^ Logic Model and a standard set of survey questions (20). The Logic Model illustrates the causal assumptions linking coalition activities to long-term, measurable outcomes. State coalition leaders can choose to focus their efforts using the logic model (adaptable to states’ specific needs) as a guide. The Logic Model articulates the relationship between the resources used to operate the coalition, the activities that the coalition conducts, and the outcomes and impact that the coalition will achieve. By demonstrating the progression, state coalitions can help stakeholders understand how their work leads to desired outcomes.

The standard set of survey questions measure progress of key stakeholders including older adults, children of older adults, primary care providers, and state legislators. The question sets were selected from validated surveys and research activities, and states were asked to use the questions as designed since the standard set of questions must be the same to allow comparisons across states and to demonstrate national impact. Three states (Kansas, New York, and New Hampshire) added a subset of questions from the standard set of survey questions to their state Behavioral Risk Factor Surveillance System (BRFSS) survey. In 2012, 94 people from across the country attended a webinar to learn how to use the Evaluation Guidelines (personal communication, Emily Dessem, National Council on Aging, 2014 March 2).

### Implementing fall prevention policy and systems change

Through these various efforts, significant progress was made in the areas of increasing fall prevention awareness, creating multidisciplinary networks, and identifying evaluation measures. However, to achieve systems change and long-term sustainability, state fall prevention coalitions recognized that implementing policy change was necessary, and subsequently requested assistance from Falls Free^®^ leadership in identifying, implementing, and advancing a full array of fall prevention policies to pursue.

**Figure 2:**
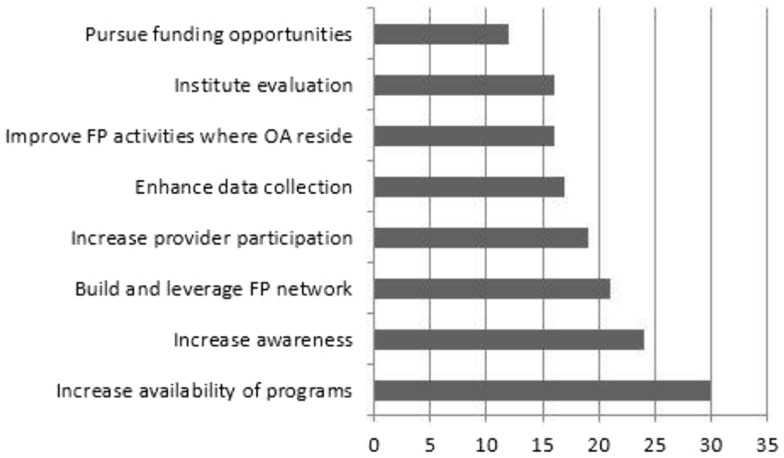
**Policy goals being “actively worked on” by state fall prevention coalitions are shown**.

To assist the states in their policy development, Falls Free^®^ leadership developed and released the *State Policy Toolkit for Advancing Falls Prevention*, which incorporates the previously discussed Falls Free^®^ Logic Model as a framework to advance policy change (21). The tool kit was disseminated to the State Workgroup on Fall Prevention Coalition members and on the NCOA website. A webinar was held in 2013 to train over 250 attendees on how to use and implement policies in the policy tool kit. A survey of state fall prevention coalition leads was conducted prior to the webinar to ascertain policy implementation, and results indicate that coalitions are actively pursuing evidence-based policies (see Figure 2).

## Discussion and Lessons Learned

Although the Falls Free^®^ Initiative played an important supportive role in the fall prevention coalition movement, progress would not have been possible without the work of thousands of people across the country, including the state fall prevention coalition leaders and members, public health community, aging services network, health care providers, researchers, and many, many others.

Creating a movement is not simple, but there are several lessons learned from the progress made thus far that could be applied to other older adult health care issues at the state or local level (11):

- Identify and promote the issue.
  - Use available data to define the issue, convey its impact, and design strategies.
  - Collect and share personalized stories of the impact of the issues and how programs and services are making a difference in the lives of older adults.
  - Advocate with legislators and decision-makers to promote your issue; if your organization is not permitted to advocate, find partners who can.
- Engage partners and leaders.
  - Find partners whose missions align in some way with your issue.
  - Choose engaged, passionate, determined leaders, and champions to promote the issue.
  - Clarify organizational relationships for lead roles and joint planning activities. Develop partnerships between health care, aging, public health, and research networks.
  - Promote collaboration in the cross-agency planning, design, implementation, and evaluation of programs for older adults.
  - Be inclusive; all stakeholders have a role to play.
- Identify solutions.
  - Seek out and adapt measurable, feasible, evidence-based solutions.
  - Increase the availability of tools, resources, and programs, so affected individuals will have methods to reduce their individual risks.
  - Keep trying if you fail. It can take time for a movement to take root, grow, and bloom.

While there has been significant progress in raising awareness of older adult falls, increasing the number of fall prevention coalitions, promoting evidence-based programs, instituting evaluation, implementing fall prevention policy and systems change, and enhancing federal funding for fall prevention efforts, much work still must be done. Older adults themselves and their caregivers need to take a more proactive role in fall prevention. Perhaps due to the stigma of falling, lack of understanding that many falls can be prevented, or limited knowledge on how to get involved, consumers are not yet well engaged in offering new goals for advancing this movement.

The escalating issue of older adult falls affects every state. As the Falls Free^®^ Initiative has demonstrated, one effective approach is an inclusive, targeted coalition to bring partners together to address the issue. A successful rallying activity is the annual observance of FPAD. However, this one observance needs to be leveraged into a more comprehensive approach to community fall prevention.

Despite the states’ request for coalition evaluation guidelines, uptake has been limited. States may not have embraced the guidelines due to lack of funding to conduct evaluations or lack of awareness that the guidelines are available.

Future research is needed to better understand how states and communities can implement policy and systems change to more effectively implement falls prevention initiatives within and across sectors such as health care, aging services, and public health. Additionally, the escalating issue of older adult falls is severely underfunded, so partners and stakeholders must continue to advocate for support.

With its growing network of dedicated champions, the Falls Free^®^ Initiative will continue its collaborative efforts to address these areas of focus with the ultimate goal of reducing the number of falls and fall-related injuries, increasing life expectancy, and improving quality of life among older adults in the United States.

## Conflict of Interest Statement

The authors declare that the research was conducted in the absence of any commercial or financial relationships that could be construed as a potential conflict of interest.

This paper is included in the Research Topic, “Evidence-Based Programming for Older Adults.” This Research Topic received partial funding from multiple government and private organizations/agencies; however, the views, findings, and conclusions in these articles are those of the authors and do not necessarily represent the official position of these organizations/agencies. All papers published in the Research Topic received peer review from members of the Frontiers in Public Health (Public Health Education and Promotion section) panel of Review Editors. Because this Research Topic represents work closely associated with a nationwide evidence-based movement in the US, many of the authors and/or Review Editors may have worked together previously in some fashion. Review Editors were purposively selected based on their expertise with evaluation and/or evidence-based programming for older adults. Review Editors were independent of named authors on any given article published in this volume.
